# The pathological and therapeutic roles of mesenchymal stem cells in preeclampsia

**DOI:** 10.3389/fmed.2022.923334

**Published:** 2022-07-28

**Authors:** Sanshan Jin, Canrong Wu, Ming Chen, Dongyan Sun, Hua Zhang

**Affiliations:** ^1^Hubei University of Chinese Medicine, Wuhan, China; ^2^Department of Traditional Chinese Medicine, Maternal and Child Health Hospital of Hubei Province, Wuhan, China; ^3^The CAS Key Laboratory of Receptor Research, Shanghai Institute of Materia Medica, Chinese Academy of Sciences, Shanghai, China; ^4^Department of Rehabilitation Physiotherapy, Maternal and Child Health Hospital of Hubei Province, Wuhan, China; ^5^Department of Gynecology, Maternal and Child Health Hospital of Hubei Province, Wuhan, China

**Keywords:** PE, MSCs, MSCs transplantation, exosome, pregnancy, placenta

## Abstract

Mesenchymal stem cells (MSCs) have made progress in the treatment of ischemic and inflammatory diseases. Preeclampsia (PE) is characterized by placenta ischemic and inflammatory injury. Our paper summarized the new role of MSCs in PE pathology and its potency in PE therapy and analyzed its current limitations. Intravenously administered MSCs dominantly distributed in perinatal tissues. There may be additional advantages to using MSCs-based therapies for reproductive disorders. It will provide new ideas for future research in this field.

## Introduction

Preeclampsia (PE) is a leading gestational disease that harms both the mother and the fetus in the short and long term. Mesenchymal stem cells (MSCs) are coordinated in endometrium decidualization and placental development. MSCs derived from PE patients show high senescence and apoptosis rate which impair the crosstalk between MSCs and endothelium, trophoblast, and immune cells in the placenta, thereby hastening the progression of PE. Preclinical and clinical data indicate that the MSCs-based therapeutic strategy is promising to be used for ischemic and inflammatory diseases. PE is characterized by placenta ischemic and inflammatory injury. MSCs have been recently applied to PE therapy. MSCs and their derivatives can ameliorate symptoms and maternal-fetal outcomes in PE model mice by boosting cell metabolism, anti-oxidative stress, stimulating angiogenesis balance, and anti-inflammation. We have summarized the interactive mechanisms between MSCs and trophoblast under physiological and hypoxic conditions in this article for the first time. MSCs therapy may show extra benefits in PE therapy for its dominant distribution in perinatal tissue. Although intravenous injection of MSCs has shown safety in clinics so far, additional research into the safety of their administration during pregnancy is needed. MSCs-derived exosome (Exo) might be a viable option for PE therapy.

## Mesenchymal stem cells

**FIGURE 1:**
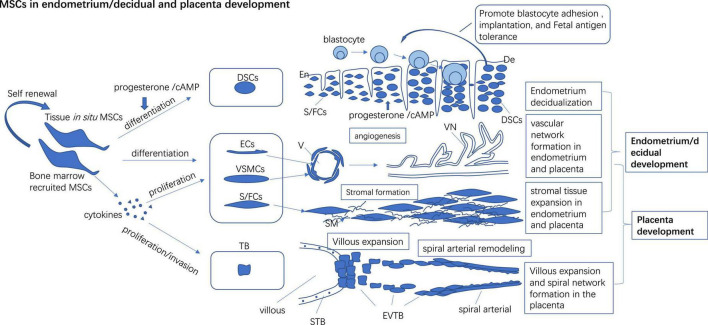
*In situ* and bone marrow recruited MSCs to participate in endometrium/decidual and placenta development by direct differentiating into endothelial, vascular smooth muscle cells, and stromal cells to form vascular and stromal tissue *de novo* and promote the proliferation of preexisting vascular cells and the stromal cells to build the vascular network and stromal in tissue. Progesterone/cAMP induces decidual stromal cell (DSCs) like changes in MSCs and expresses high levels of receptive markers which may promote blastocyte adhesion, implantation, and fetal antigen tolerance. MSCs secret multiple cytokines to promote proliferation and invasion of trophoblast (TB) which may foster villous expansion and maternal spinal arterial remodeling. En, Endometrium; De, Decidual; Ecs, Endothelial cells; VSMCs, Vascular smooth muscle cells; S/FCs, Stromal/fibroblast cells; V, Vascular; VN, Vascular network; SM, Stromal matrix; STB, Syncytiotrophoblast; EVTB, Extravillous trophoblast.

Synchronized endometrium decidualization, embryo implantation, and sufficient placentation are necessary for a successful pregnancy. Although lineage hierarchy and cell fate in the placenta and decidual are not well defined so far, it is undeniable that the whole endometrium regeneration, decidualization, and placentation process are mainly driven by diverse programmed stem cell activities (trophoblast stem cells, MSCs, epithelial progenitors, endothelial progenitors, etc.). Typical MSCs give rise to endothelial and vascular smooth muscle-like cells (they all participate in forming the vascular wall) in conditional mediums and form tube-like structures in Matrigel (Figure 1). MSCs secrete free or Exo encapsulated small molecules (such as cytokines, RNA and DNA) participating in various signal transduction to surrounding cells. It is well-known that MSCs have trophic effects on surrounding cells by secreting growth factors like vascular endothelial growth factor (VEGF), hepatocyte growth factor (HGF), placenta growth factor (PGF), etc. MSCs also act as atypical immune cells participating in immune modulation. Until now, it is still difficult for us to get the *in situ* dynamic spatio-temporal data about how the stem cells actually build the endometrium and placenta. Despite its limitation in representing *in situ* conditions, stem cell *in vitro* differentiation, gene mapping, transcriptome, and secretome evaluation are still key media for understanding how MSCs work.

### Mesenchymal stem cells in endometrium regeneration

Mesenchymal stem cells in the endometrium have similar functions to bone marrow-derived MSCs (bm-MSCs). Endometrium MSCs (eMSCs) have specific markers like CD146+, SUSD2+ (sushi domain containing-2), and PDGFRb+ (platelet-derived growth factor receptor beta), which characterized their perivascular location and possible pericyte identity (1). Besides, stromal fibroblast in the endometrium also has some MSCs properties and multilineage differentiation potential *in vitro* (1). Masuda found that eMSCs can differentiate into endometrial stromal structures following xenografting under the kidney capsule in the immune-compromised mice (2). *In situ* transplantation of MSCs can rebuild the endometrium of women with thin endometrium and restore the embryo implantation rate of these patients (3, 4).

### Mesenchymal stem cells in decidualization

Endometrium experiences a transient decidualization stage which confers a special micro-environment to accept the implantation of the conceptus, regulate the invasion of trophoblast, and induce immune tolerance to fetal antigen (5–7) (Figure 1). Decidualization disorder is not only related to embryo implantation failure but also other lagging pregnancy disorders like miscarriage, PE, intrauterine growth restriction (IUGR) (8, 9).

Decidual stromal cells (DSCs) are the hallmark cell of the decidualization process. Recent studies identified that DSCs precursor cells (preDSCs) and eMSCs similar to each other phenotypically (CD140b, CD146, and SUSD2) and functionally (10, 11). Progesterone and cAMP primed the decidualization process (12). Progesterone (P4) and cAMP can induce decidualization-like changes in eMSCs *in vitro* [eMSCs become bigger and round, lose perivascular cell markers, and express prolactin (the marker of DSCs)]. Thus, eMSCs and preDSCs were postulated to be two closely related cell types or even the identical one type (13). High levels of endometrium receptivity markers like Integrin (ITG)β1, ITGβ3, Rac1, Noggin, and Homeobox A11 (HOXA11) (14) and other genes involved in inflammation, immunomodulation, hypoxia responses, and cell communication are detected in decidual MSCs (dMSCs). These changes may furnish the decidual with a friendly environment for embryo adhesion, implantation, and placentation (1) (Figure 1). Upregulated expression of insulin-like growth factor (IGF), transforming growth factor β (TGFβ), Notch, and Hedgehog receptor signaling pathway genes suggest high self-renewal and differentiation of MSCs in decidual (1). Low levels of receptivity markers were detected in dMSCs from women with recurrent implantation failure (RIF) (14). Some functional disorders have also been detected in PE-derived dMSCs and we have discussed them intensively in the following paragraphs.

### Mesenchymal stem cells in placentation

**FIGURE 2:**
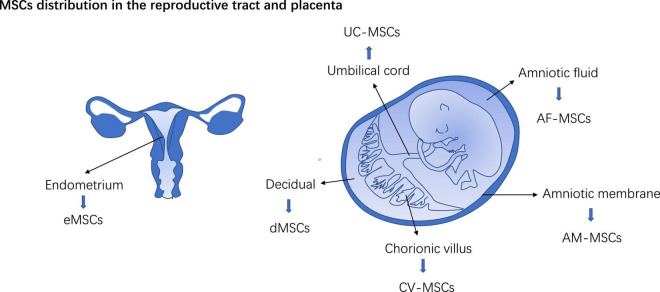
MSCs distribution in the reproductive tract and placenta. eMSCs, Endometrium MSCs; dMSCs, decidual MSCs; AM-MSCs, Amniotic membrane-derived MSCs; UC-MSCs, Umbilical cord MSCs; AF-MSC, Amniotic fluid MSC; CV-MSCs, Chorionic villus-derived MSCs.

Mesenchymal stem cells in perinatal tissue were classified into dMSCs, amniotic membrane-derived MSCs (AM-MSCs), umbilical cord MSCs (UC-MSCs), amniotic fluid MSC (AF-MSC), and chorionic villus-derived MSCs (CV-MSCs) according to their tissue site in the placenta (Figure 2). In the early stage of pregnancy, the embryo doubles in size per week thus both trophoblasts and mesenchyme in the placenta expand at a rapid rate (15). MSCs from the early trimester placenta possess the ability to differentiate into tissue from three germ layers (16). PD-MSCs show high clone formation capacity and pro-angiogenesis potency (17). In *in vitro* co-cultivation system, PD-MSCs foster the functions of the trophoblast and educate immune cells in the placenta (mentioned later). Reshef Ta had discerned a small population of bm-MSCs in mice placenta, they express progesterone receptor (PR), a hallmark of decidualized stromal cells (7). Hoxa11 deficiency leads to pregnancy loss in mice (18). In the bone marrow, Hoxa11 does not express in hematopoietic cells but bm-MSCs (19). Transplantation of bone marrow from Hoxa11+/+mice can favor embryo implantation and rescue pregnancy loss in Hoxa11 ± mice (7). Maria Diniz-da-Costa characterized a transient group of highly proliferative bone marrow-derived MSCs (hPMC) in the implantation window in women’s endometrium, loss of hPMC was detected in women with recurrent pregnancy loss (20). It posits that bm-MSCs recruited to the decidual and placenta may participate in maintaining a normal pregnancy.

## Preeclampsia

Preeclampsia is a disease that affects approximately 5–7% of pregnancies (21, 22). PE is a multifactorial and multiorgan syndrome leading to maternal and neonatal morbidity. PE is characterized by proteinuria and hypertension and often occurs after 20 weeks of gestation (23). By now, the etiology and pathogenesis of PE are gradually elucidated (24). After embryo implantation, ectodermal cells differentiate into trophoblast cells and invade maternal uterine tissue, transform maternal uterine blood vessels, and form a stable maternal-fetal blood supply network, the placenta. The placenta is a highly vascularized temporary organ responsible for nutrients and metabolic waste exchange between mother and fetal through the placenta blood supply. Superficial trophoblast invasion, insufficient placenta formation, or other factors affecting local blood vessels of the placenta will lead to impaired placental blood perfusion, resulting in placental and fetal hypoxia/ischemia injury.

Hypoxia/ischemia related mitochondrial dysfunction (25), endoplasmic reticulum stress (26), autophagy/mitophagy disorder induce tissue oxidative injury, and finally cell apoptosis or pyroptosis in the placenta (27). High level of damage-associated molecular patterns (DAMPs) (28), pro-inflammatory cytokines, combined with increased level of anti-angiogenic factors [soluble endoglin, soluble FMS-like tyrosine kinase-1 (sFlt-1)] (29, 30) release from the injured placenta into circulation to provoke local and systematic inflammation and endothelial dysfunction (24, 31, 32). Multiple systems are implicated in PE thus it manifests as hypertension, proteinuria, retinal edema, liver and kidney dysfunction, or even life-threatening HELLP syndrome (33).

## Mesenchymal stem cells dysfunction in preeclampsia

Recent studies have found that MSCs dysfunction is associated with the pathogenesis of PE. Human UC-MSCs (hUC-MSCs) obtained from PE women showed elevated ROS levels, decreased telomerase activity, and elevated expression of senescence-related genes (DEGs) (24, 34). JunB and Cyclin-D1 are key cell cycle-related modulators. JunB/Cyclin D1 imbalance in PE placenta-derived MSCs (PD-MSCs) can block the G1/S cell cycle transition resulting in cell senescence and reduced proliferation (35, 36). MSCs dysfunction may impair the self-proliferation and differentiation of MSCs in the PE placenta and also its crosstalk with trophoblast cells, immune cells, and endothelial cells (37–39), which may further speed up the progression of PE. Although it is not clear whether decidual/placenta MSCs dysfunction is the cause or the sequence of PE, MSCs dysfunction may promote the vicious cycle of PE.

Oxidative stress injury and inflammatory condition of the placenta may be partly responsible for impaired MSCs function in PE patients. Though moderate hypoxia can stimulate MSCs to resist oxidative stress injury, and promote angiogenesis and its self-renewal (40, 41), intensive hypoxia can lead to MSCs senescence and apoptosis (42). In addition, as inflammatory factors excite the immunomodulatory traits of MSCs, they also trigger the apoptosis of MSCs (43, 44). A variety of miRNAs and long non-coding RNAs (LncRNAs) abnormally expressed in PE patients mediate MSCs dysfunction (45, 46). These RNAs regulate genes involved in cell proliferation, senescence, apoptosis, immune/inflammation modulation, and the angiogenesis process of MSCs (47) (Table 1). Heme oxygenase 1 (HMOX1) is a multifunctional stress-response protein and plays anti-oxidant (48–50), anti-apoptosis, and anti-inflammation roles in tissues (51). HMOX1 remains at a high level throughout gestation, but Basmaeil

**TABLE 1:** miRNA and LncRNA involved in MSCs dysfunction in PE.

MSCs kinds	miRNA levels in PE	miRNA biotargets	Molecules involved	miRNA bioeffects on MSCs	References
dMSCs	miR-136↑	\	VEGF↓	**dMSCs:** cell proliferation ↓, apoptosis↑; angiogenesis↓ **dMSCs on trophoblasts:** invasion ↓; **dMSCs on HUVECs:** capillary formation↓.	(147)
hUC-MSCs	miR-495↑	Bim-1	\	**hUC-MSCs:** inhibit cell proliferation↓, senescence↑, apoptosis↑, migration↓, invasion↓; **hUC-MSCs on trophoblasts:** migration↓, invasion↓; **hUC-MSCs on HUVECs:** capillary formation↓.	(78)
hUC-MSCs	miR-181a↑	\	CD450↓, IL-6↑, VEGF↑, IDO↑, CD8^+^/IFN-γ positive T-cells↑	**hUC-MSCs:** proliferation↓; **hUC-MSCs on T-cells:** proliferation and activation↓.	(148)
hUC-MSCs-Exo	miR-30a↑	TAB3	TAB3↓, cyclin E 2↓, p-IkBα/IkBα↓, P-JNK/JNK↓, IL-6↓, IL-8 ↓, COX2 ↓	**hUC-MSCs:** cell cycle entry rate↓; **hUC-MSCs on Treg:** the induction of CD4^+^CD25^+^Foxp3^+^ Treg cells↓; **hUC-MSCs on Macrophage:** TNF-α↑, IL-6↑.	(112)
dMSCs	miR-16↑	\	cyclin E1↓ VEGF-A↓	**dMSCs:** proliferation↓, migration↓, cell-cycle arrests↑; **dMSCs on trophoblast:** migration↓; **dMSCs on HUVECs:** capillary formation↓.	(80)
dMSCs	miR-494↑	\	CDK6↓, CCND1↓, cyclin D2 (CCND2)↓, cyclin E1↓, VEGF↓	**dMSCs:** cell cycle at G1/S stage↑; **dMSCs on trophoblast:** migration↓; **dMSCs on HUVECs:** capillary formation↓.	(79)
hUC-MSC-Exo	miR-133b↓	SGK1	SGK1↑, Cyclin D1↓, Ki-67↓, Bcl-2↓ Bax↑	**hUC-MSCs on trophoblast**: cell cycle progression↑, apoptosis↓, proliferation↑, migration ↑, invasion↑.	(68)
Bm-MSCs	lncRNA H19↓	let-7b	let-7b↓, FOXO1↑, p-FOXO1↑, AKT↑, p-AKT↑, VEGF↑, IDO↑, CD14^+^/CD206^+^ macrophages↑	**Bm-MSCs on trophoblasts:** migration↑, invasion↑, apoptosis↓	(149)
hUC-MSCs	lncRNA MALAT1↓	\	VEGF↑, IDO↓	**hUC-MSCs:** proliferation↑, apoptosis↓, migration↑, invasion↑; **hUC-MSCs on trophoblasts**: migration↑, invasion↑; **hUC-MSCs on HUVECs:** capillary formation↑; **hUC-MSCs on Macrophage:** MSCs induced macrophage M2 polarization↑.	(114)
hUC-MSC-Exo	MiR-101↓	BRD4	BRD4↓, NF-KB↓, CXCL11↓, IL-6↓, TNF-α↓, p65↓, p-lkBα↑	**hUC-MSC: Trophoblasts:** migration↑, proliferation↑.	(149)
hUC-MSC-Exo	miRNA-139-5p\	PTEN	↑PTEN↓, c-caspase-3↓, p-ERK1/2 ↑, MMP-2↑	**hUC-MSC on trophoblasts:** migration ↑, invasion ↑, apoptosis↓.	(118)
hUC-MSC-Exo	miRN- 18b↓	Notch2	Notch2↓, TIM3↓, mTORC1↓	**hUC-MSC on trophoblasts:** proliferation↑, migration↑.	(150)

TAB 3, Transforming growth factor-β-activated kinase 1 binding protein 3; SGK1, Serum and glucocorticoid-inducible kinase 1; BRD4, Bromodomain-containing protein 4; PTEN, Phosphatase and tensin homolog.

Y. found HMOX1 decreased in one subtype of PD-MSCs in PE patients. H_2_O_2_ preconditioning upregulate HMOX1 expression and restore the function of PD-MSCs, but is nullified by tin protoporphyrin, an HMOX1 selective inhibitor (37). HMOX1 deficiency may be partly responsible for PD-MSCs dysfunction in PE patients. TNF-α is a pro-inflammatory cytokine elevated in PE. TNF-α induces a senescent phenotype of adipose-derived MSCs (adMSCs) with strong staining for senescence-associated components (52). Dasatinib, a senolytic agent, significantly rescues senescence and restores the proliferation and angiogenesis potency of adMSCs from PE patients (52, 53). Compared to the normal placenta, PE-derived PD-MSCs secrete higher levels of pro-inflammatory cytokines like IL-8, IL-6, migration inhibitory factor (MIF), TNF-α (36). PD-MSCs extracted from PE patients impede VEGF and β-human Chorionic Gonadotropin (βhCG) expression and stimulate a high level of sFlt-1 secretion to form a PE like phenotype in normal term villous explants (36).

It was newly proved that human CV-MSCs (hCV-MSCs) are abundant with primary cilium (54). They are responsible for cell signaling, differentiation, motility, and homing. Abnormal cilium length was detected in PE-derived hCV-MSCs. It may cripple the differentiation of hCV-MSCs and its interaction with other cells such as trophoblast and endothelial cells in the placenta (HUVECs), followed by the impaired capacity of hCV-MSCs to foster the growth of human placental organoids and vascular-like network formation of Human Umbilical Vein Endothelial Cells (HUVECs) *in vitro* (54).

## Mesenchymal stem cells in preeclampsia therapy

Besides responding to growth signals and participating in programmed tissue development, MSCs also act as damage sensors and are recruited to the injury site in response to stress signals (inflammation, hypoxia, and the like) and then take part in tissue repair (55). MSCs replenish injured tissue by both differentiating into tissue cells directly and secreting trophic cytokines to foster the proliferation of tissue cells indirectly. Moreover, inflammatory signals can also stimulate MSCs to an immunosuppressive phenotype thus curbing inflammation/immune activation in injured tissue (43). MSCs-mediated angiogenesis help injured tissue to rebuild the vascular network and recover blood supply. More interestingly, MSCs express low levels of HLA Class I and II and high levels of HLA-G (56, 57), as a consequence, MSCs show relative low immunogenicity after allotransplantation. Paracrine nutrition, multi-differentiation, damage sensing, anti-inflammation/immune-modulation, and low immunogenicity traits have made MSCs transplantation a potent therapy in tissue repair and systematic inflammation/immune disorders (58). Japan and Europe have approved MSCs products for the clinical treatment of Crohn’s fistular and graft vs. host diseases (59). Now, MSCs transplantation becomes a new remedy realized in PE-like animal models.

### Mesenchymal stem cells and trophoblasts

#### Superficial trophoblast invasion in preeclampsia

During placentation, trophoblasts invade the decidual and form the villous, the base functional unit of the placenta. Extravillous trophoblast (EVT) migrates out from the villi to fix to the decidual. Other endovascular EVT invade the spiral arteries to replace the vascular wall (60) and enhance the placental blood supply to the fetus (61, 62). Superficial trophoblast invasion and insufficient placental vascular remodeling were detected in PE patients (33, 63, 64) leading to placental hypoxia/ischemia in PE (65, 66).

#### Mesenchymal stem cells and trophoblast function

**FIGURE 3:**
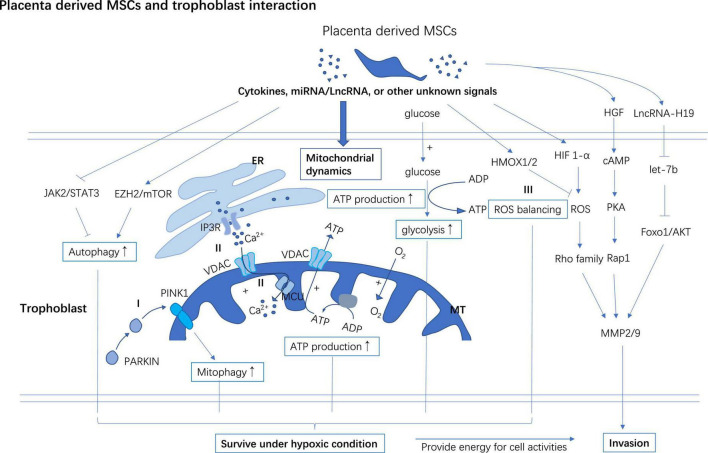
PD-MSCs support trophoblast invasion and improve survival of trophoblast in hypoxia. PD-MSCs secret multiple cytokines, miRNA, LncRNA, or other undefined signals to facilitate the invasion of trophoblast. PD-MSCs primed autophagy and mitophagy may help trophoblast survive under hypoxic conditions. PD-MSCs promote mitochondrial ATP synthesis through glycolysis and oxidative phosphorylation which increase the energy supply for trophoblast activities and survival under hypoxic conditions. **(I)** After preconditioned in a hypoxic environment, PD-MSCs co-cultivation improves PINK1 accumulation in MT, and then PINK1 interacts with PARKIN to induce mitophagy in trophoblast. **(II)** PD-MSCs promote Ca^2+^ commuting between ER and MT through activating IP3R/MCU in the trophoblast. **(III)** PD-MSCs upregulate HIF-1α in trophoblast followed by a moderate surge of ROS flux to activate the invasion ability of trophoblast, however, PD-MSCs also promote the expression of HMOX1/2 in trophoblast to curb excessive oxidative injury induced by ROS. ER, Endoplasmic reticulum; MT, Mitochondrial; EZH2, Zeste 2 polycomb repressive complex 2; IP3R, Inositol 1,4,5-trisphosphate; MCU, Mitochondrial calcium uniporter; VDAC, Voltage-dependent anion-selective channel; PINK1, Phosphatase and tensin homolog (PTEN)-induced kinase 1; PARKIN, Parkin RBR E3 ubiquitin-protein ligase.

Mesenchymal stem cells derived Exo promote migration, invasion, and proliferation of trophoblasts cell lines *in vitro*, promoting cell cycle entry and inhibiting apoptosis of these cell lines (67, 68). Both hUC-MSCs and its supernatant can upregulate PGF and β-hCG levels in the HTR8-S/Vneo culture medium and promote the proliferation, migration, and invasion of HTR8-S/Vneo trophoblasts cell line (69). It was reported that the invasiveness of trophoblast is partially regulated by paracrine signaling from PD−MSCs (70). The HGF generated from PD−MSCs promotes trophoblast invasion by upregulating cAMP and Rap1 (71) (Figure 3).

Hypoxia-inducible factor 1-alpha (HIF1-a) is a multifunctional transcription factor involved in regulating energy metabolism, cell survival, invasion, and angiogenesis. HIF1-α is a regulator of ROS to stimulate cell invasion and migration by activating (72) the ERK (73), and Rho (74) family signaling pathways. MMP-2/-9 facilities the invasion process of trophoblast by breaking down the extracellular matrix. It has been found that PD-MSCs promote trophoblasts invasion partially by upregulating HIF1a mRNA and MMP-2/-9 mRNA (74) (Figure 3).

In addition, miRNAs and LncRNAs in MSCs derived Exo MSCs are transferred to the trophoblast to regulate its activities. LncRNA-H19, LncRNA-MALAT, miR-101, miR-133b, and miR-18b in MSCs-Exo promote trophoblast proliferation, migration, and invasion *in vitro* (Table 1). But these miRNAs and LncRNAs are abnormally expressed in PD-MSCs derived from PE patients (Table 1). For example, LncRNA-H19 in MSCs-Evo targets let-7b to up-regulate FOXO1 to activate the AKT signaling pathway thus increasing invasion/migration and inhibiting apoptosis of trophoblast cells (75) (Figure 3). Another LncRNA named metastasis-associated lung adenocarcinoma transcript 1 (MALAT1) is first discovered to prophesy lung cancer metastasis (76, 77) now found to be involved in promoting trophoblast proliferation and invasion. However, low levels of H19 and MALAT1 were detected in hUC-MSCs from PE patients (78). miR-16 and miR-494 inhibit the migration of trophoblast, while they are both up-regulated in PE-derived MSCs (79, 80). After treatment with PE-derived PD-MSCs conditioned media (CM), disturbed JunB/Cyclin D1 balance combined with arrested cell cycle and elevated production of pro-inflammatory cytokines were detected in normal term placental villous explants *in vitro* (39).

#### Mesenchymal stem cells regulate autophagy in the trophoblast

Autophagy is important to maintain homeostasis in humans. Lysosomes remove degradation molecules, invading pathogens, and malfunction organelles through autophagy (81). Under hypoxia, ischemia, starvation, or other stress conditions, cells get nutrients and energy partially through activating autophagy (82, 83). Molecules involved in the autophagy process also participate in cell proliferation, differentiation, and senescence signaling pathways (84). Autophagy is crucial for placentation (85). In Syncytio-trophoblasts, autophagy protects the cell from infection, apoptosis, and inflammation (86, 87). In hypoxic/ischemic conditions, placental trophoblasts in PE patients are more reliant on autophagy to survive than cells in the normal placenta (88).

Modulating autophagy is one of the mechanisms involved in the therapeutic effects of MSCs in injury tissue repair (89). MSCs can modulate autophagy in immune cells and facilitate the resolution of injury-related inflammation (89). In addition, MSCs-mediated autophagy promotes the survival, proliferation, and differentiation of tissue stem/progenitor cells to support the restoration of the functional tissue after injury (89). BM-MSC activates the autophagic machinery and promotes the survival of pulmonary cells in ischemia-reperfusion-injury models *in vivo* and *in vitro* (90). This effect was also detected in trophoblast (91, 92).

JAK2/STAT3 (92) is the putative upstream regulator activating autophagy while mTOR/Zeste 2 polycomb repressive complex 2 (EZH2) subunit axes are putative to down-regulate autophagy in cells (93–95). Under the hypoxic condition, AD-MSCs-Exo and CV-MSC conditioned medium boost autophagy, invasion, and survival of trophoblasts by inhibiting the EZH2/mTOR and activating the JAK2/STAT3 signaling pathway in the trophoblast respectively (91, 92) (Figure 3). Blocking the JAK2/STAT3 signaling pathway or stimulating the expression of EZH2 or administration of autophagy inhibitor 3-MA can reduce MSCs cultivation mediated autophagy, invasion, and survival of trophoblasts (91, 92).

#### Mesenchymal stem cells regulate mitochondrial metabolism

High-energy cells like trophoblast rely intensely on mitochondrial ATP synthesis to function normally and then establish a successful pregnancy. PD-MSC cocultivation improves glycolysis and mitochondrial respiration (74). It improves cellular ATP synthesis and consumption *via* activating the Ca^2+^ movement between the endoplasmic reticulum (ER) and mitochondria in invasive trophoblasts and significantly increases trophoblasts’ invasion ability (74). Inositol 1,4,5-trisphosphate (IP3R) in ER interacts with mitochondrial calcium uniporter (MCU) and voltage-dependent anion-selective channel (VDAC) (an ion channel in the outer membrane of mitochondria) and plays a role in calcium transportation (96–98) (Figure 3). Cellular levels of MCU, VDAC, and IP3R all increased in trophoblast after PD-MSCs co-cultivation (74) (Figure 3).

In PE patients, placenta hypoxia/ischemia-induced oxidative stress results in mitochondria dysfunction and then cell apoptosis. Several studies showed smaller mitochondria and increased ROS levels in trophoblast extracted from the placenta of women with PE, indicating a mitochondrial malfunction in trophoblast cells (99).

Hypoxia brings on disarrangement of mitochondrial ultrastructure in mice trophoblasts *in vitro*. bm-MSCs intensify mitochondrial membrane potential and increase ATP production/consumption in trophoblast and support cell survival against hypoxia (100). PD−MSCs reduce mitochondrial damage by downregulating Heat shock protein 60 (HSP60) (inducted under mitochondrial stress) and upregulating prohibitin 1 (PHB1) (involved in stabilizing mitochondria) expression and promoting ATP generation/consumption by upregulating VDAC in the mitochondria of trophoblast cells (101).

ROS is generated dynamically in cell metabolism or under stress conditions, and it is degraded by antioxidant enzymes to keep ROS at an appropriate level in mitochondria. ATP production/consumption and oxidative phosphorylation (OXPHOS) synchronously increase with the invasiveness of cells (102). Mitochondrial ROS (mtROS) promotes cell migration *via* the NADPH oxidase (NOX) signaling pathway (102). Moreover, mtROS signal promotes structure stability of MMP9 mRNA to upregulate cell invasiveness (103). PD-MSCs co-cultivation activates the HIF1-α/ROS/MMPs stream to promote the invasion of trophoblast through the ERK signaling pathway (73). While, excessive ROS accumulation impairs the invasiveness of trophoblasts through downregulating integrin b3 and FOXO1 (104, 105). HMOX1/2 are genes targeting ROS to manage oxidative stress. After cocultured with PD-MSCs, HMOX1/2 mRNA and protein levels were increased in trophoblasts compared to un-cocultured ones. These data indicate that MSCs play a role in balancing ROS to facilitate trophoblast invasion while avoiding a high level of ROS-mediated cell injury (74, 101) (Figure 3).

#### Mesenchymal stem cells regulate mitophagy in the trophoblast

Mitochondrial autophagy (mitophagy) is a self-protection mechanism of cells under stress, which is usually triggered by damaged mitochondrial. Persistent hypoxia induces mitophagy to clear the damaged mitochondria and keep cellular homeostasis (106). Phosphatase and tensin homolog (PTEN)-induced kinase 1 (PINK1) and Parkin RBR E3 ubiquitin-protein ligase (PARKIN) are two mitophagy regulators. It was reported that PINK1 modulates mitochondrial metabolism, and calcium homeostasis (107). PINK1 binds with PARKIN to recognize the proteins on the outer membrane of mitochondria and mediate autophagy to remove the damaged mitochondria. PD-MSCs cocultivation up-regulates the expression of PINK1 and PARKIN in the trophoblast and protects trophoblast survival from hypoxia (101, 108) (Figure 3).

In all, MSCs promote the proliferation and function of trophoblast cells *via* the paracrine pathway, and these effects were attenuated in PE-derived MSCs. By facilitating mitochondrial metabolism, stabilizing the mitochondrial membrane, and modulating the autophagy and mitophagy in the trophoblast, MSCs promote the survival of the trophoblast and maintain its proliferation, migration, and invasion under hypoxic conditions. MSCs supplementation may help restore the trophoblast function in the hypoxic placenta in PE patients.

### Mesenchymal stem cells in inflammation/immune modulation

Local and systematic inflammation along with excessive immune activation was detected in PE gestation. The inflammatory process mediates placenta and systematic vascular endothelial injury and angiogenesis disorder in PE. Persistent hypoxia-related oxidative stress induces high amounts of DAMP generated in the placenta, which activate the innate and adaptive immune systems. Neutrophils, monocytes, NK cells, DCs are all possible objective cells involved in inflammation conditions in PE (109).

Mesenchymal stem cells is a stress sensors. Under the inflammatory condition, MSCs cell-to-cell contact with macrophages, monocytes, dendritic cells (DCs), natural killer (NK) cells, T cells, and B cells as well as release paracrine cytokines like prostanoid E2 (PEG2), indoleamine 2,3-dioxygenase (IDO), TGF-β1, HGF, and nitric oxide (NO) to regulate innate and adaptive immunity (43).

**FIGURE 4:**
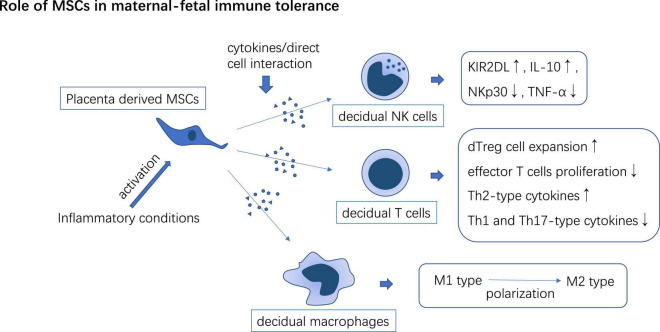
PD-MSCs interact with decidual-derived immune cells to form immune tolerance phenotype which may participate in maternal-fetal immune tolerance induction.

Mesenchymal stem cells are involved in regulating the immune response in decidual. dMSCs upregulate KIR2DL1, and IL-4, and downregulate the expressions of NKp30 and TNF-α thus inducing a tolerance phenotype of dNK cells (110). After co-culture with immune cells isolated from decidual tissue, UC-MSCs promote the expansion of decidual Treg cells (dTreg), inhibit effector T cells proliferation, enhance Th2-type cytokines secretion in T cells, and enhance the potency of dTreg to suppress Th1 and Th17 mediated inflammation (111). MSCs secrete TSG-6 or cell-to-cell contact with pro-inflammatory macrophages through CD200/CD200R axis to educate macrophages toward an anti-inflammatory phenotype. The immunomodulatory effects of MSCs are usually activated in inflammatory conditions (43). MSCs may contribute to the transition of the early stage Th1 inflammation state in the maternal-fetal interface to the immune tolerance state in the second trimester of pregnancy (Figure 4).

miRNA/LncRNA expression disorder in patients with PE can disturb the immune-modulatory function of MSCs in the placenta (Table 1). MiR-30a and miR-494 are up-regulated in hUC-MSCs derived from PE patients. IL-1β-pretreated hUC-MSCs significantly reduce lipopolysaccharide (LPS) elicited IL-6 and TNF-a expression in macrophages and facilitate CD4^+^CD25^+^Foxp3^+^ Treg cells expansion, but miR-30a transfection impairs these anti-inflammatory effects of hUC-MSCs through targeting at transforming growth factor-β-activated kinase 1 binding protein 3 (TAB3) (112). miR-494 reduces PGE2 secretion in decidual MSCs (dMSCs) and attenuates PGE2 mediated M2 macrophage (anti-inflammatory phenotype) polarization *in vitro* (113). MALAT1 induces IDO expression in UC-MSCs and promotes M2 macrophage polarization *in vitro*, however, the level of LncRNA-MALAT1 decreased in UC-MSCs in patients with severe PE (114).

Mesenchymal stem cells have immune-modulatory and anti-inflammatory potency thus they have been used in a variety of inflammation-related diseases, such as graft vs. host disease and Crohn’s disease (115). MSCs or MSCs-EVs were also found to have *in vivo* anti-inflammatory effects in N-nitro-L-arginine methyl ester (L-NAME) (116), LPS (117), endotoxin (118), or Th1 cell-induced PE-like mouse model (119). Intravenous administration of MSCs or MSCs-Exo reduces inflammatory cytokines such as TNF- α, IL-1 β, and IL-6 in PE mice on the contrary increasing IL-10 and PPAR γ levels in it (Table 2). In addition, experiments also confirmed that MSCs had a direct effect on LPS-induced trophoblast inflammation *in vitro*. After LPS pretreated trophoblast cocultured with AF-MSC, miR-146a-5p was upregulated in trophoblast cells. miR-146a-5p in AF-MSCs derived Exo suppress the inflammatory pathway like NFκB and MAPKs in trophoblast after treatment with LPS (120). This may be one of the mechanisms by which MSCs promote the survival of trophoblasts in PE-related placental inflammatory environments.

**TABLE 2:** MSCs and its derivants transplantation in PE-like animal models.

MSCs kinds	Animals	PE-like modeling	Route of MSCs administration	Bioeffects of MSCs administration	References
hUC-MSCs	Rat	LPS (i.v.)	2 × 10^6^ cells per mouse (i.v.) once	BP↓, TNF-α↓, IL-6↓, IL-12↓, ICAM-1↓, IL-10↑.	(100)
hdMSCs	Mice	activated Th1 cells (i.v.)	100 μl (10^6^ cells/100 ml, i.v.) on day 11.5 and day 13.5 of gestation	BP↓, UP↓, fetal weight↑, fetal loss rate↓, TNF-α↓, placental and glomerular injury↓.	(73)
hUC-MSCs	Rat	endotoxin solution (i.v.)	100 μl (2 × 10^6^ cells/100 μl, i.v.) once	BP↓, UP↓, TNF-α↓, IL-1β↓, IL-10↑	(151)
HMOX1 modified hPD-MSCs	Rat	L-NAME (i.p.)	25 μl (5 × 10^7^) injected into placenta	BP↓, UP↓, placental and fetal weight↑, placental perfusion↑, placental angiogenesis balance ↑ (MVD↑, VEGF↑, and PlGF↑, sFlt-1↓, and sEng ↓).	(137)
hUC-MSC-Exo	Rat	L-NAME (i.p.)	0.5 ml/rat/day (120 μg/ml, i.p.), totally 6 days	BP↓, UP↓.	(94)
hUC-MSCs-EVs	Rat	L-NAME (s.c.)	Not detailedly mentioned	BP↓, UP↓, apoptotic cell rate in placenta↓.	(150)
hUC-MSC-Exo	Rat	L-NAME (i.p.)	0.5 ml/rat/day (i.v., low (120 μg/ml), middle (140 μg/ml), high (160 μg/ml) level), totally 6 days	BP↓, UP↓, fetal numbers↑, placental and fetal weight↑, apoptotic cell rate in placenta↓, placental angiogenesis balance ↑(MVD↑, VEGF↑, sFlt1↓).	(138)
hUC-MSC-EVs	Mice	HMOX1 null mice model	5 × 10^6^ cell equivalents (i.v.) once	BP↓, UP↓, fetal loss rate↓, fetal length↑, placental and kidney injury↓, placental spiral artery lumen:wall ratio↑, placental uNK and myeloid cell numbers↑, CD44, CD103, and CD64 level in myeloid populations↑, IL-10 and IFN-γ↑.	(152)
hPD-MSCs-CM	Mice	LPS (i.v.)	300 μL (i.v.) once	BP↓, UP↓, placental weight↑, sFlt-1↓, IL-6↓, and TNF-α↓.	(153)
hUC-MSCs-Exo	Rat	L-NAME (i.p.)	20 μl /rat/day (80 μg/20 μl, i.p.) on day 16 to day 19 of gestation	BP↓, UP↓, fetal and placenta weights↑, TNF-α↓, IL-1β↓, IL-6↓, apoptotic cell rate in placenta↓.	(116)
MiR-101 modified hUC-MSC-EVs	Rat	L-NAME (i.p.)	140 μg/ mL since the 14th day of pregnancy for 6 days (i.p.)	BP↓, 24 h-UP↓, fetus and placental weights↑, placenta injury↓, CXCL11↓, IL-6↓, TNF-α↓, p65↓, p-IkBα↑.	(149)

L-NAME, NG-nitro-L-arginine methyl ester; i.p., intraperitoneal injection; i.v, intravenously injection; BP, blood pressure; UP, urine protein.

### Mesenchymal stem cells and oxidative stress in preeclampsia

As mentioned earlier, MSCs participate in the antioxidant stress process by promoting trophoblast autophagy, regulating mitochondrial metabolism, promoting mitochondrial autophagy, and balancing ROS levels, to induce trophoblast survival in hypoxic conditions. Aldehyde dehydrogenases (ALDH) are enzymes detoxifying aldehydes generated under oxidative stress. Immunohistochemical localization found that ALDH was co-localized with the FZD-9 (a specific MSCs marker) in maternal *decidua basalis.* MSCs derived from the placenta show high ALDH activity under oxidative stress (121). ALDH1A1 mRNA level and ALDH enzyme activity are decreased in PE dMSCs relative to normal dMSCs. PE-derived dMSCs have an impaired response to oxidative stress with increased ROS levels in them (122). Moreover, PD-MSCs secreted paracrine factors trigger STAT3 activation and superoxide dismutase 2 (SOD2) production to support endothelial cell survival under tert-Butyl hydroperoxide induced oxidative injury (123). dMSCs significantly enhance the activities of glutathione and thioredoxin reductases in H_2_O_2_ preconditioned HUVECs and restore their function (124). In addition, dMSC-EVs significantly reduce the level of lipid peroxidation in PE serum treated HUVECs (125).

### Mesenchymal stem cells in angiogenesis

Mesenchymal stem cells are involved in both vasculogenesis and angiogenesis processes *in vitro*. Pluripotent mesenchymal cells differentiate into multiple cell lineages like endothelial cells and smooth muscle cells to constitutively form vascular *de novo* (defined as vasculogenesis) (126) (Figure 1). PD-MSCs secrete various angiogenic agents like VEGF, and HGF which promote preexisting endothelial progenitor cell migration, promotion, tube formation, and sprouting to form a stable vessel network and regulate angiogenesis under the stimulation signals like hypoxia and growth (127) (defined as angiogenesis) (Figure 1). PD-MSCs exhibited superior pro-angiogenesis potential compared to bm-MSCs and UC-MSCs (128). Endometrial and gestational tissue-derived MSCs have strong therapeutic angiogenesis in clinical and experimental use (129). However, the angiogenesis potency of PD-MSCs from PE patients was compromised.

Angiogenic imbalance is one of the key steps in the pathogenesis of PE. Antiangiogenic protein sFlt-1 is elevated in the placenta and serum of PE patients. sFlt-1 binds to VEGF and PGF to inhibit their proangiogenic process (130). sFlt-1/VEGF imbalance leads to endothelial dysfunction and angiogenic disorder in patients with PE (131, 132). The sFlt-1/VEGF ratio has emerged as a key biochemical indicator for predicting the risk of PE (113). Treating villous explants from normal placenta with PE-derived PD-MSCs conditioned media, the villous showed significantly increased expression of sFlt-1 and decreased VEGF protein level compared to the normal PD-MSCs group (36).

PD-MSCs and their derived EVs promote HUVECs tube formation *in vitro*. HMOX1 is a stress-response protein with pro-angiogenic properties (133–135). HMOX1 modified PD-MSCs show higher efficiency than the unmodified ones in promoting HUVECs tube formation (136). In the L-NAME-induced PE-like rat model, HMOX1 modified PD-MSCs restore VEGF/sFlt-1 balance to form a proangiogenic state *in vivo* and increase microvascular density (MVD) in the placenta (137). Intravenous administration of hUC-MSCs-Exo could significantly alleviate endothelial nitric oxide synthase induced placental angiogenesis disorder, and increase VEGF level and placental MVD in pregnant rats (138).

## Limitations and prospects

Among the 11 PE animal pieces of research (Table 2), including 4 MSCs-based studies and 7 MSCs derivates (6 MSCs-Exo and 1 MSCs-CM) based studies. MiR-101 transfection and HMOX1 gene-modifying enhance the efficacy of hUC-MSC-EVs and hPD-MSCs in PE mice therapy, respectively. MiR-18b-3p, MiRNA-101, and MiR-139-5p are molecules in MSCs-Exo partially responsible for relieving symptoms and improving pregnancy outcomes in PE mice. One study found dose-dependent therapeutic effects of MSC-Exo on PE mice. How PE modeling, MSCs cell types, and administration routes influence therapeutic efficacy in PE mice can’t be concluded from these data, and also, no systematic experimental design was conducted to evaluate the toxicity of MSCs administration during pregnancy.

Bone marrow, adipose, and perinatal tissues are important sources of MSCs. The special phenotype of MSCs from different tissues affects their safety and efficiency in treatment. In recent years, more and more attention has been paid to the clinical application of perinatal tissue-derived MSCs. Many shreds of evidence support that MSCs from gestational tissue show lower aging rates and higher proliferation efficiency (56), superior regenerative and immunosuppressive activities in some clinical and preclinical studies (56). Meanwhile, placental neonate-derived MSCs express lower levels of HLA class I and II and higher levels of HLA-G, which may reduce the risk of immune rejection in clinical application (56). The unique growth and immune microenvironment of MSCs in the placenta may explain these traits in perinatal tissue-derived MSCs. All studies included in Table 2 have chosen perinatal tissue-derived MSCs in PE mice therapy.

Efficacy and safety are two major concerns in MSCs therapy. Scientists are trying to solve the problems of automatic differentiation, loss of stemness, and senescence of MSCs during cultivation and administration. MSCs apoptosis and immune rejection result in a short half-life of MSCs *in vivo*. Intravenously administered MSCs going through lung arrest and only a little of it homing to the injury site. These may explain the limited therapeutic potency of MSCs *in vivo*.

Implantation cytokine (IFN-τ) and embryonic trophectoderm secretomes chemotaxis peripheral blood MSCs and adult bone marrow progenitors toward the uterus (7, 139, 140). MSCs are actively recruited to decidual and contribute to embryo implantation (7, 139, 140) and may also play role in controlling embryo implantation provoked inflammation because inflammatory signals activate the anti-inflammatory and immunomodulatory potencies of MSCs (141). *In vivo*, optical data shows that intravenous transferred Zs-Green^+^ MSCs were predominantly distributed in the pregnant uterus than in the virgin uterus in mice and increased in LPS-induced inflammatory pregnant uterus in a further step. The advantageous biological distribution of MSCs in pregnant tissues may encourage MSCs in treating pregnancy diseases (141). Though MSCs have low immunogenicity, allogeneic MSCs transplants may also induce immune rejection. Transplanted MSCs die rapidly then it be cleared by innate immune cells (142, 143). Whether the immune-privileged maternal-fetal interface will benefit the survival of allogeneic MSCs is an interesting perspective to investigate.

Mesenchymal stem cells have shown high safety in clinical and experimental studies, but there are still several important issues that need to be treated carefully because safety is the most basic prerequisite for the application of any kind of treatment. Although MSCs do not have a direct tumorigenic effect, MSCs have the risk of promoting tumor growth in tumor patients (144). Trophoblast cells have biological characteristics similar to those of tumor cells. Large-scale experimental observation is needed to determine whether MSCs will increase the risk of abnormal invasion of trophoblast or even choriocarcinoma. MSCs treatment is associated with an increased risk of thrombosis (145). It is open to debate whether MSCs adoption further raises the risk of thrombosis under preexisting physiologic hypercoagulation state during pregnancy, especially in the third trimester of pregnancy.

Exo is naturally generated nanosized vesicles containing growth factors, cytokines, lipids, regulatory miRNAs, and DNA. These vesicles comprise natural lipid bilayers embed with an abundance of adhesive proteins and readily interact and fuse with cellular membranes (146). Exo is involved in cell-to-cell signaling communication at short or long distances and can respond to tissue injury, infection, and disease. MSCs-Exo is with many traits of MSCs in the treatment of hypoxia-induced tissue injury (146). MSCs-Exo also has therapeutic effects on PE as to current mice data, and it shows lower immunogenicity and higher therapeutic safety, thus MSCs-Exo will be a good substitute for MSCs. Meanwhile, we may further improve the therapeutic effect of MSCs and MSCs-Exo through gene modification strategies.

## Author contributions

SJ was responsible for the data collation and full-text writing of this article. MC, CW, DS, and HZ were responsible for the proofreading of this article. HZ also funded this article. All authors contributed to the article and approved the submitted version.

## Conflict of interest

The authors declare that the research was conducted in the absence of any commercial or financial relationships that could be construed as a potential conflict of interest.

## Publisher’s note

All claims expressed in this article are solely those of the authors and do not necessarily represent those of their affiliated organizations, or those of the publisher, the editors and the reviewers. Any product that may be evaluated in this article, or claim that may be made by its manufacturer, is not guaranteed or endorsed by the publisher.
